# Mapping of C5‐Blocking Monoclonal Antibodies Reveals New C5 Inhibitory Epitopes and Novel Modes of C5 Inhibition

**DOI:** 10.1111/imm.70180

**Published:** 2026-07-27

**Authors:** Rebekah Sian Cooke, Kok Yung Lee, Georgina Elizabeth Menzies, Masashi Mizuno, Bryan Paul Morgan, Wioleta Milena Zelek

**Affiliations:** ^1^ UK Dementia Research Institute Cardiff, School of Medicine Cardiff University Cardiff UK; ^2^ Molecular Biosciences Division, School of Biosciences Cardiff University Cardiff UK; ^3^ Department of Renal Replacement Therapy Nagoya University Graduate School of Medicine Nagoya Japan

**Keywords:** C5, complement system, crovalimab, eculizumab, mechanism, membrane attack complex, therapeutic antibodies

## Abstract

C5 inhibition is a proven therapeutic target with several monoclonal antibody (mAb) and small molecule drugs in the clinic. To explore alternative modes of inhibition, novel C5‐blocking mAbs were generated and compared to therapeutic mAbs eculizumab and crovalimab. Blocking mAbs were selected using complement inhibition in haemolysis assays. C5 binding and competition between mAbs were tested by ELISA and surface plasmon resonance. Impact on C5 cleavage by CVF and native convertases was tested by western blotting of C5 fragments and ELISA for C5a release. Four novel C5‐blocking mAbs were identified and shown to inhibit haemolysis with comparable efficacy to crovalimab and eculizumab. Competition assays identified four distinct function‐blocking epitopes; mAb 7D4 and eculizumab competed for an α‐chain epitope (epitope 1), while 10B6 and crovalimab competed for a β‐chain epitope (epitope 2). mAbs 2B11 and 4G2 were not competitive with epitope 1/2 binders or each other, marking two distinct blocking α‐chain epitopes (epitopes 3 and 4). Epitope 1 and epitope 2 binders blocked C5 convertase‐mediated C5 cleavage and C5a release, while epitope 3 and epitope 4 binders permitted C5 cleavage, demonstrating distinct modes of inhibition. We identified four distinct C5 blocking epitopes; epitope 1 targeted by eculizumab and 7D4, epitope 2 targeted by crovalimab and 10B6 and epitopes 3 and 4 targeted by 2B11 and 4G2 respectively. Although all efficiently blocked haemolysis, epitope 3 and 4 binders permitted C5a release. We reveal (at least) four blocking epitopes on C5 and (at least) two distinct mechanisms of inhibition.

## Graphical abstract

We identify four distinct C5 inhibitory epitopes, including two novel antibodies that selectively block membrane attack complex (MAC) assembly while preserving C5 cleavage and C5a generation. These findings establish selective MAC inhibition as a new therapeutic strategy for complement‐mediated diseases.

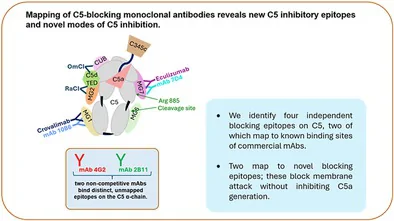

## 1 Introduction

Complement therapeutics has evolved from limited applications in a few ultra‐rare conditions to the application of complement inhibition to more common diseases [1]. C5 emerged early as a favoured therapeutic target; indeed, the first human C5‐blocking monoclonal antibody (mAb) was reported in 1991 [2, 3]. Progress toward the clinic was spearheaded by the C5 blocking mAb eculizumab, FDA approved for paroxysmal nocturnal haemoglobinuria (PNH) in 2007. The complement therapeutics landscape has recently expanded considerably, including seven FDA‐approved agents targeting C5 or its downstream effector C5a, with numerous others in development [4, 5, 6]. Among these, crovalimab stands out with its extended half‐life enabled by sequential mAb recycling technology (SMART). Eculizumab binds the C5 α‐chain in the MG7 domain [7], while crovalimab binds the C5 β‐chain; both inhibit C5 cleavage, preventing MAC assembly and C5a generation [8, 9]. This shared mechanism despite divergence in binding sites highlights the structural and functional complexity of C5 inhibition and opens new avenues for further investigations.

Here we compared four in‐house C5‐blocking mAbs, 2B11, 4G2, 7D4 and 10B6 [10], with the clinically validated C5‐blocking therapeutic mAbs eculizumab and crovalimab to define mechanisms of action, identify distinct functional epitopes on C5 and determine competition between the mAbs. We demonstrate additional blocking epitopes on C5 distinct from those occupied by eculizumab and crovalimab and identify a novel C5 inhibitory mechanism: prevention of MAC assembly without preventing C5 cleavage and generation of C5a. This unique mechanism of MAC‐specific inhibition offers an alternative approach to therapy of MAC‐driven disease and may inform the design of next‐generation complement therapeutics tailored for specific diseases.

## 2 Methods

All chemicals were of analytical grade and from either Sigma Aldrich (Gillingham, UK) or Fisher Scientific (Loughborough, UK) unless otherwise stated. Eculizumab was donated by Professor David Kavanagh (Newcastle, UK); crovalimab was donated by Roche Diagnostics (Basel, Switzerland). Serum from a patient expressing the R885H C5 variant was provided by Prof Masashi Mizuno (Nagoya, Japan) [11]. Samples were collected under appropriate institutional ethics approval with written informed consent from all participants in accordance with local regulations and the Declaration of Helsinki. Normal human serum (NHS) was prepared in‐house from freshly collected blood donated by healthy volunteers. C5, C5b6, C7, C8 and C9 proteins were purified from NHS using previously described immunoaffinity methods [9, 12]. The C5‐inhibiting mAbs 4G2, 7D4 and 10B6 were described previously but not epitope‐mapped or functionally characterised [10]. The anti‐C5 mAb 2B11 was generated from a different fusion and not previously reported. The four anti‐C5/C5b6 mAbs further characterised (4G2 and 7D4, IgG2bκ; 10B6, IgG1κ; 2B11, IgG2bκ) recognised C5 and/or C5b6 but not C6. 4G2 and 7D4 bound the C5 α‐chain, whereas 10B6 and 2B11 recognised the C5 β‐chain [10]. 4G2 and 7D4 inhibited complement activation in human, rat and rabbit sera, with weaker activity against guinea pig and mouse sera, whereas 10B6 and 2B11 were specific for human complement.

### 2.1 Enzyme‐Linked Immunosorbent Assays (ELISAs)

To test binding of mAbs to C5, MaxiSorp (Nunc, Loughborough, UK) 96‐well plates were coated with C5 (0.5 μg/mL in carbonate buffer pH 9.6) overnight at 4°C. Plates were washed using phosphate‐buffered saline (PBS) with 0.05% (v/v) tween‐20 (PBS‐T), blocked using 2% (w/v) bovine serum albumin (BSA) in PBS‐T for 1 h at 37°C, and washed again. Test mAbs were titrated (2–0 μg/mL) in 0.2% BSA‐PBS‐T and incubated for 1 h at 37°C followed by washing. Horseradish peroxidase (HRP)‐conjugated donkey antimouse IgG (for in‐house mAbs; Jackson ImmunoResearch, #715‐035‐150) or donkey antihuman IgG (for humanised mAbs; Jackson ImmunoResearch, #709‐036‐146) secondary antibodies were added (1:2000 in 0.2% BSA‐PBS‐T) for 1 h at 37°C. Plates were washed and developed using sigma‐fast O‐phenylenediamine dihydrochloride (OPD) tablets (Sigma Aldrich, #P9187) in the dark, the reaction halted using 5% (v/v) sulphuric acid, and absorbance measured at 492 nm using an Infinite F10 plate reader (Tecan).

Sandwich ELISAs were used to determine competition for C5 binding between mAb pairs. For capture, individual mAbs were coated at 1 μg/mL overnight at 4°C and plates were blocked as above. C5 protein was titrated from 2 to 0 μg/mL in 0.2% BSA‐PBS‐T and incubated 1 h at 37°C, followed by washing. For detection, biotinylated mAbs (EZ‐link‐sulfo‐biotin as per manufacturer's instructions; Thermo Fisher Scientific, #A39257) were added at 1 μg/mL in 0.2% BSA‐PBS‐T and incubated for 1 h at 37°C. After washing, streptavidin‐HRP (R&D Systems, #890803) was added at 1:200 in 0.2% BSA‐PBS‐T and incubated for 1 h at 37°C. Plates were washed and developed as described above.

To compare binding of tested mAbs to C5 in NHS and R885H variant serum, plates were coated with goat antihuman C5 (1.5 μg/mL; Complement Technology, #A220) as capture and blocked with 3% nonfat milk (NFM) in PBS‐T. Serum samples (1:1000) or purified C5 standards diluted to generate a standard curve (0.63–0 μg/mL) were added in duplicate, incubated for 1 h at RT and washed. Test mAbs were then added at 2 μg/mL in 0.3% NFM in PBS‐T and incubated for 1 h at RT, followed by washing. Bound mAb were detected using donkey antimouse‐HRP for mouse mAbs and donkey antihuman F(ab')₂‐HRP for the human mAbs (Jackson ImmunoResearch; 715‐035‐150, 709‐036‐149), diluted 1:3000 in 0.3% NFM in PBS‐T and incubated for 1 h at RT. Plates were washed and developed using TMB and absorbance measured at 540 nm. C5 concentrations were calculated from the linear portion of the ELISA standard curve, corrected for dilution and expressed as μg/mL.

A commercial C5a ELISA (R&D Systems, #DY2037) was used, as instructed by the manufacturers, to measure C5a in supernatants from the CH50 and CVFBb cleavage assays described below. In brief, MaxiSorp 96‐well plates were coated with capture antibody in PBS at RT overnight. Plates were blocked using 1% BSA‐PBS for 1 h and washed in PBS‐T. Samples were added at 1:4 (for CH50 supernatant) or 1:400 (for CVFBb reaction) in triplicate in BSA‐PBS for 2 h at RT, washed and incubated with biotinylated detection mAb for 2 h. After washing and incubation with streptavidin‐HRP (1:200) for 30 min at RT, plates were washed, developed using TMB, absorbance measured at 540 nm. Concentrations were calculated from the linear portion of the standard curve.

To assess activation of the terminal pathway, terminal complement complex (TCC) was measured. Plates were coated overnight at 4°C with aE11 mouse anti‐human TCC mAb (Hycult Biotech HM2017) at 5 μg/mL in bicarbonate buffer, blocked for 1 h at 37°C using 2% BSA‐PBS‐T and washed as described above. CH50 supernatant was added at 1 in 2 in 0.2% BSA‐PBS‐T for 1 h at 37°C followed by washing and 1 h incubation at 37°C with anti‐C8 mAb E2‐biotin at 1 μg/mL (in‐house). Following washes, streptavidin‐HRP (R&D Systems #890803) was added at 1:200 in 0.2% BSA‐PBS‐T, incubated for 1 h at 37°C then washed and developed as described above. Concentrations were calculated from the linear portion of the standard curve.

### 2.2 Haemolytic Assays

The capacity of each of the mAbs to inhibit C5 and prevent MAC formation was tested in classical pathway haemolysis assays (CH50). A 2% suspension of sheep erythrocytes (ShE; from sheep blood in Alsever's solution, TCS Biosciences) in HEPES‐buffered saline (HBS) was prepared then sensitised for complement activation by incubating with anti‐ShE antiserum (Amboceptor, 1:4000; ORLC25, Cruinn Diagnostics, Dublin, Ireland) for 30 min at 37°C [13]. Unless otherwise stated, mAbs were tested at a final concentration of 50 μg/mL. This concentration was selected based on preliminary optimisation experiments to ensure maximal complement inhibition while allowing direct comparison between mAbs across human complement assays. The sensitised ShE (ShEA) were washed and resuspended at 2% in HBS. NHS dose for ~80% lysis was assessed by titration for each ShEA batch (typically, 5%). To titrate complement inhibition, mAbs were serially diluted from 200 to 0 μg/mL in 96 well plates (50 μL) then NHS at the 80% lysis dose and 2% ShEA (50 μL each) added and incubated for 30 min at 37°C. The reaction was stopped by centrifugation at 800*g* for 5 min at 4°C and supernatant transferred to flat bottom plates for measurement of haemoglobin release at 405 nm. Percentage lysis was calculated as (sample—negative control)/(positive control—negative control) ×100. To assess ability of mAbs to inhibit lysis with C5 R885H variant serum, CH50 assays were performed using 100 μg/mL mAbs with 5% R885H serum.

Terminal pathway inhibition was assessed using reactive lysis assays as described [9, 14, 15]. Guinea pig erythrocytes (GpE, TCS Biosciences) were diluted to 1.5% (v/v) in HBS and added to a 96 well round bottom plate (50 μL/well). In‐house purified components (12.5 μL/well) were added sequentially with a 10 min incubation (37°C) between components and no wash steps: C5b6 (final conc. 45 ng/mL), C7 (final conc. 11.5 ng/mL), C8 (final conc. 84 ng/mL), C9 (final conc. 383 ng/mL), followed by a final 30 min incubation at 37°C, centrifugation and measurement of lysis as above. Inhibitors were added at one of three stages to assess their MAC blocking mechanism: prior to C5b6 addition, prior to C7 addition, or prior to C8/C9 addition. Irrelevant mAb D1.3 was used as a negative control throughout. Plates were centrifuged and haemolysis measured as above.

### 2.3 CVF Convertase Inhibition Assay

The CVFBb C5 convertase was generated by mixing cobra venom factor (CVF; purified in‐house from *Naja naja kaouthia* venom as described [16]), factor B (FB; affinity purified in‐house) and factor D (FD; A136, Comptech, USA) in a 1:1:0.1 molar ratio and incubating for 1 h at 37°C. C5 was incubated with each of the mAbs (5:1 molar excess of mAb) at RT for 30 min, CVFBb added (1:5 CVFBb:C5 molar ratio) and incubated for 1 h in a 37°C shaking water bath. A portion of the reaction mixture was retained for measurement of C5a in the ELISA and the remainder diluted 1:3 in HBS and processed for western blot analysis as described below.

### 2.4 Western Blotting

Samples were resolved by SDS‐PAGE under reducing (R) conditions on 7.5% bis‐tris gels (run for 30 min at 180 V in 1× NuPAGE MOPS running buffer (Invitrogen, #NP0001)). For western blotting, gels were transferred onto nitrocellulose membranes for 1 h at 10 V in an Invitrogen miniblot transfer system then stained with ponceau S to confirm successful transfer and loading quality. Membranes were blocked using 5% (w/v) BSA PBS‐T for 1 h at RT, probed with anti‐C5a mAb (2 μg/mL; clone 2942, Hycult, #H2078; Uden, Netherlands) overnight at 4°C, washed in PBS‐T then incubated for 1 h at RT with donkey antimouse IgG‐HRP (Jackson ImmunoResearch, #715–035‐150) diluted 1:10000 in 5% BSA‐PBS‐T. Membranes were washed in PBS‐T, developed using chemiluminescence (ECL Substrate, Cytiva, Amersham, RPN2209), and imaged on a G:BOX XX6 (SynGene).

For additional western blot analyses, C5 protein was resolved by SDS–PAGE under R and NR conditions, transferred to nitrocellulose membranes, probed with the different anti‐C5 mAbs, detected and developed as described above.

### 2.5 Affinity Measurement and Epitope Binning Using Surface Plasmon Resonance

The C5 binding kinetics of mAbs: 4G2, 7D4 and 10B6 have been reported previously (Table 1) [10]. The KD for mAb 2B11 was measured using surface plasmon resonance (SPR; Biacore T200, Cytiva). The mAb was immobilised in the appropriate orientation using a Mouse IgG Fc capture kit (Cytiva, #BR100838), unbound mouse IgG capture sites blocked by flowing 500 nM mouse IgG (purified from mouse serum on a protein G column; Cytiva, #17040501). C5 was then flowed at different concentrations and the KD calculated using the inbuilt software, calculated using the Langmuir 1:1 binding model with RI values set to zero.

**TABLE 1:** A summary of binding affinities (KD) of anti‐C5 mAbs.

mAb	KD (M)
4G2	1.389 × 10^−8^
7D4	1.264 × 10^−9^
10B6	4.105 × 10^−10^
2B11	5.02 × 10^−10^
Crovalimab	1.66 × 10^−10^
Eculizumab	~1.2 × 10^−10^

*Note:* The table summarises the kinetic data for C5 binding across the panel of in‐house (4G2, 7D4, 10B6 and 2B11) and commercial (crovalimab and eculizumab) [9, 17] C5 mAbs. Primary kinetic data for the mAb 2B11 are in Figure S2.

Epitope binning of mAbs was performed via SPR. Individual mAbs were immobilised in the appropriate orientation using the Mouse IgG Fc capture kit as above by flowing at 100 nM over the chip for 4 min, followed by mouse IgG to block residual sites. C5 at 100 nM was flowed over the immobilised mAb for 2 min, then each of the mAbs was individually flowed at 100 nM for 2 min to assess binding to the captured C5. Following each cycle, the chip was regenerated using 10 mM glycine (pH 1.7) for 5 min and the cycle of IgG capture, C5 binding and second mAb binding was repeated.

The human mAbs eculizumab and crovalimab were not compatible with the mouse IgG capture kit. To test these, the mAbs were preincubated in molar excess with C5 protein and the mAb‐C5 complexes flowed over the immobilised mAbs. Failure to bind the mAb‐C5 complex indicated competition for the same site on C5 while binding indicated noncompetitive interactions. The cycle was repeated for each pair of in‐house and therapeutic mAbs and data were analysed as described above.

To confirm the presence of multiple, distinct mAb‐binding epitopes on C5, a further SPR ligand binding assay was performed; mAb 2B11 (100 nM) was immobilised using the mouse IgG capture kit by flowing over the chip, followed by blocking as described above. mAb 7D4 (100 nM) preincubated with an equimolar amount of C5 protein was flowed over the 2B11 surface to capture the complex. mAbs 10B6 and 4G2 were flowed sequentially with no regeneration between injections to reveal all available epitopes. The human mAbs were not analysed in this experiment because the Biacore evaluation software is limited to four ligands per analysis. Their epitope relationships were therefore assessed manually by comparing binding responses (RU) calculated from the sensorgrams.

### 2.6 Measuring Formation and Decay of Terminal Complement Complexes via Surface Plasmon Resonance

To interrogate the effects of C5‐binding mAbs on C5b‐9 formation, we first immobilised eculizumab on the chip; eculizumab was CAP‐tagged using the CAP conjugation kit (Cytiva, #29805822) according to the manufacturer's instructions, then immobilised on a Series S sensor chip CAP flow cell 4 (Cytiva, #29805820) for 120 s, leaving flow cell 3 blank as a reference. Immunoaffinity purified terminal complement proteins C5b6, C7, C8 and C9 were sequentially flowed over both flow cells at 50 nM for 120 s per injection. To investigate the impact of the mAbs on binding and complex formation, each mAb (100 nM) was flowed over both flow cells for 180 s after C5b6 capture and before flowing C7. The surface was regenerated by flowing 6 M guanidine‐HCl and 0.25 M sodium hydroxide over the surface for 120 s. A new eculizumab capture surface was made as described above after every regeneration.

### 2.7 Statistical Analysis

Statistical analyses were performed using GraphPad Prism version 11.0.1 (GraphPad Software, Boston, MA, USA). Data are presented as mean ± SEM unless otherwise stated. Each independent experiment represents a biological replicate, while duplicate or triplicate measurements within an experiment represent technical replicates, as indicated in the figure legends. Statistical analyses performed for individual experiments are described in the corresponding figure legends. Comparisons between two groups were performed using an unpaired two‐tailed Student's *t* test, while comparisons among multiple groups were analysed by one‐ or two‐way ANOVA followed by the appropriate multiple‐comparison test (Tukey's or Dunnett's). Dose–response curves from haemolysis inhibition assays were analysed by nonlinear regression using a four‐parameter logistic model to calculate IC50 values with 95% confidence intervals. SPR (surface plasmon resonance) analysis was performed using Biacore T200, and binding kinetics were analysed using Biacore Evaluation Software (Cytiva) by global fitting to a 1:1 Langmuir binding model. Western blot band intensities were quantified by densitometry using ImageJ. Exact *p* values are reported where appropriate. A *p* < 0.05 was considered statistically significant.

## 3 Results

### 3.1 All mAbs Bind C5 and Inhibit Haemolysis

The in‐house mAbs 4G2, 7D4 and 10B6 were previously shown to inhibit haemolysis in CP assays and bind C5 in western blots, direct ELISA and SPR analyses [10]; this was replicated here for all these mAbs, together with the novel mAb 2B11 and the commercial mAbs eculizumab and crovalimab. IC50 values were calculated via interpolation: 2B11; 5.20 nM, 4G2; 7.47 nM, 7D4; 7.89 nM, 10B6; 3.95 nM, crovalimab; 1.96 nM, eculizumab; 2.28 nM (Figure 1A–D). Reactive lysis assays were performed for all mAbs. Eculizumab and mAb7D4 failed to inhibit lysis of GpE when added at any point in this assay initiated using preformed C5b6, confirming that eculizumab inhibits by preventing C5 cleavage, circumvented in reactive lysis and showing that mAb7D4 shares this mechanism of action [17]. In contrast, crovalimab and the mAbs 2B11, 4G2 and 10B6 all inhibited reactive lysis when added to GpE prior to or after addition of C5b6 and prior to addition of C7, but not when added after C7 incubation to form C5b‐7 sites, demonstrating that these mAbs all bound preformed C5b6 to prevent C7 binding (Figure 1E).

**FIGURE 1:**
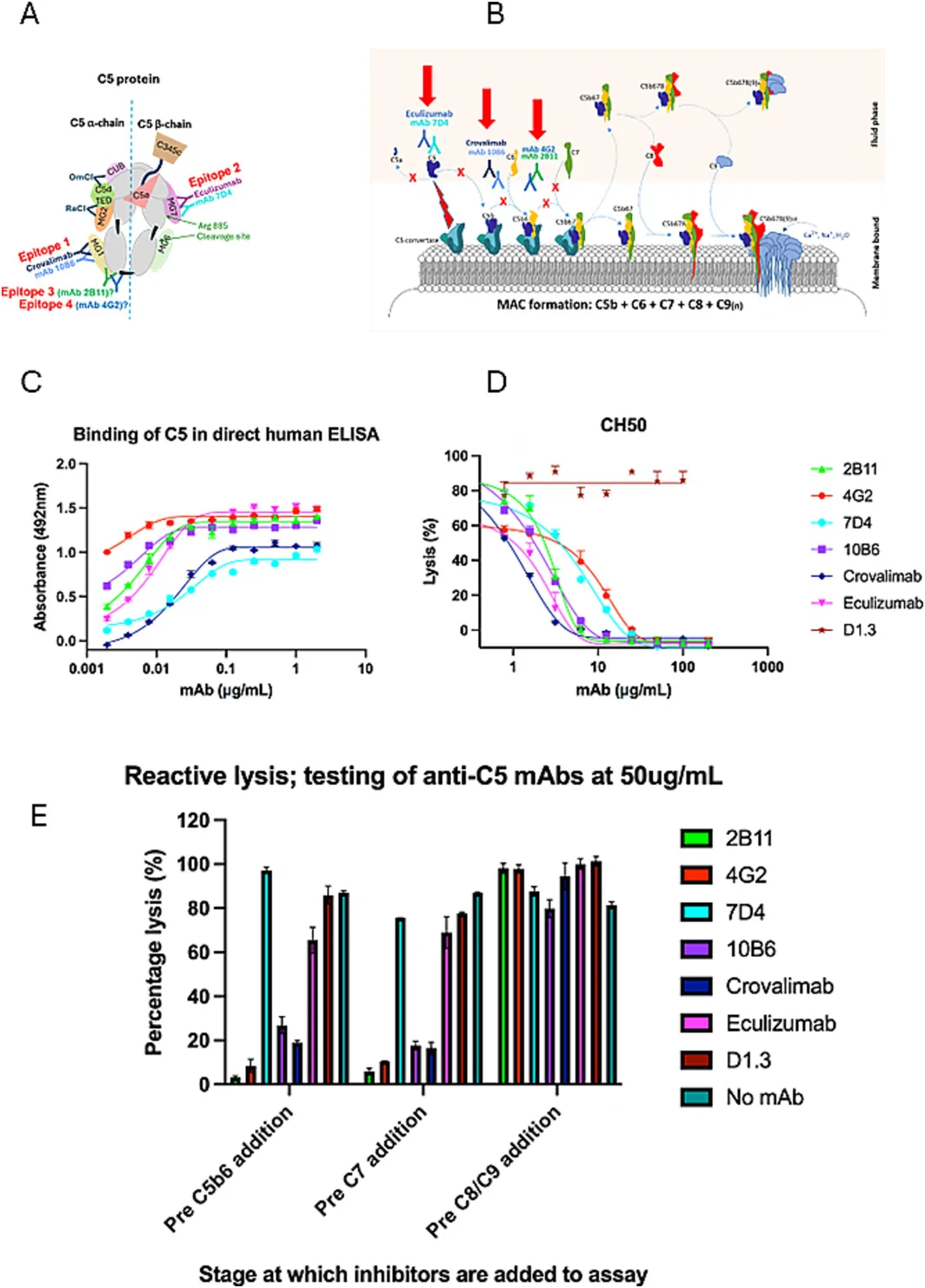
Characterisation of anti‐C5 monoclonal antibodies using direct ELISA, classical pathway haemolysis assay (CH50) and reactive lysis (RL), and summary of their epitopes and mechanisms of action. (A and B) Summary of C5 inhibitory epitopes. The shared epitopes recognised by eculizumab/7D4 (Epitope 1) and crovalimab/10B6 (Epitope 2), and the novel epitopes recognised by 2B11 (Epitope 3) and 4G2 (Epitope 4) are shown. Eculizumab, 7D4, crovalimab and 10B6 inhibit C5 cleavage, preventing both C5a generation and membrane attack complex (MAC) formation, whereas 2B11 and 4G2 selectively inhibit MAC assembly by preventing C7 binding to C5b6 while preserving C5 cleavage and C5a generation. (C) mAbs were tested for binding to immobilised human C5 in a direct ELISA; mAbs were titrated and bound mAb detected as described in methods. All tested mAbs dose‐dependently detected C5. Results are representative of three independent experiments yielding similar results. (D) mAbs were tested for inhibition of classical pathway haemolysis in CH50 assays with a serum dose selected to give ~80% haemolysis in the absence of mAb. The mAbs were titrated and haemolysis measured as described in methods. All tested mAbs dose‐dependently inhibited lysis in the classical pathway assay, with the in‐house mAbs performing similarly to the commercial mAbs eculizumab and crovalimab. Results are representative of three independent experiments yielding similar results. Error bars are standard error of the mean (SEM) of biological duplicates. IC50 values were calculated from the haemolysis inhibition curves as follows: 2B11; 5.20 nM; 4G2; 7.47 nM; 7D4; 7.89 nM; 10B6; 3.95 nM; crovalimab, 1.96 nM; eculizumab, 2.28 nM. (E) mAbs terminal pathway inhibition was tested using RL assays with the inhibitors added at 50 μg/mL either prior to C5b6 addition, prior to C7 addition or prior to C8/C9 addition. Controls included irrelevant control mAb D1.3 and no mAb. Lysis controls included HBS buffer (zero) and deionized water (100%). Results are representative of three independent experiments yielding similar results. Error bars are SEM of assay duplicates.

Western blotting confirmed that the commercial mAbs eculizumab and crovalimab bound the C5 α‐chain and β‐chain respectively; the in‐house mAbs 7D4, 4G2 and 2B11 bound the α‐chain, while mAb 10B6 bound the β‐chain (Figure S1). Binding affinities for the commercial anti‐C5 mAbs eculizumab and crovalimab (KD = ~1.2 × 10^−10^ and 1.66 × 10^−10^ respectively) [8, 17] and in‐house mAbs 4G2, 7D4 and 10B6 (KD = 1.39 × 10^−8^, 1.26 × 10^−9^, 4.10 × 10^−10^ respectively) were from our previous report [10]. SPR analysis demonstrated that mAb 2B11 bound C5 with high affinity (KD = 5.02 × 10^−10^, Table 1; Figure S2).

### 3.2 Epitope Binning and Competition Studies Identify Four Blocking Epitopes on C5

Competition between anti‐C5 mAb pairs was tested in sandwich ELISAs. When C5 was captured on either 2B11 or 4G2, all other mAbs bound captured C5, demonstrating that 2B11 and 4G2 each bound a distinct and unique epitope on C5 (Figure 2A,B). When C5 was captured on 7D4, all the mAbs except eculizumab bound, demonstrating that 7D4 and eculizumab shared a common epitope (Figure 2C), a finding replicated in the reverse format with eculizumab as capture and 7D4 detect (Figure 2F). When C5 was captured on 10B6, all mAbs except crovalimab bound, demonstrating that 10B6 and crovalimab shared a common epitope (Figure 2D), again replicated in the reverse format (Figure 2E). These results collectively demonstrate that there are at least four distinct and nonoverlapping blocking epitopes on C5: 7D4 and eculizumab binding the same epitope on the α‐chain (epitope 1), 10B6 and crovalimab binding a shared epitope on the β‐chain (epitope 2), while 2B11 and 4G2 bound distinct epitopes on the α‐chain (epitopes 3 and 4). To confirm these findings, SPR epitope binning was conducted. Neither 2B11 (Figure 3A) nor 4G2 (Figure 3B), when used as capture, showed any competition with the other mAbs or each other, confirming that each bound a unique epitope on C5. When 7D4 was used as capture, all mAbs except eculizumab bound captured C5 (Figure 3C), confirming that 7D4 and eculizumab competed for binding to C5. When 10B6 was used as capture, C5 binding was observed for all mAbs except crovalimab (Figure 3D), confirming that these mAbs shared a binding site on C5. Eculizumab and crovalimab could not be captured on the mouse IgG binding surface; instead, these mAbs were preincubated in excess with C5 then flowed over each in‐house mAb captured on the chip. The C5‐eculizumab complex bound all immobilised mAbs except 7D4 (Figure 3E), while the C5‐crovalimab complex bound all mAbs except 10B6 (Figure 3F), confirming that these mAb pairs targeted the same or closely related epitope on C5.

**FIGURE 2:**
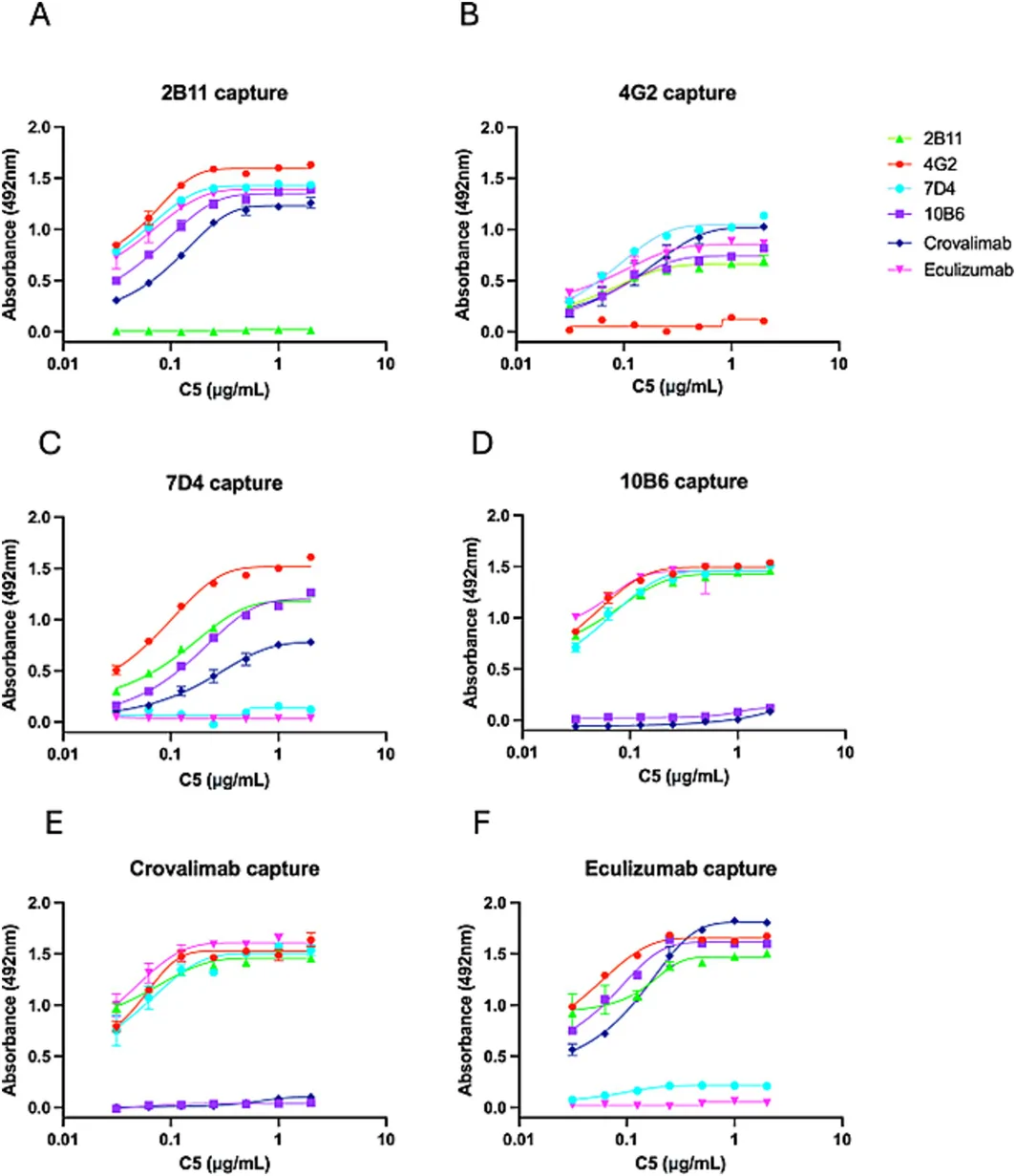
Sandwich ELISA for assessment of competition for C5 binding epitopes. (A) The mAb 2B11 was immobilised on the ELISA plate to capture C5. When added as detect, mAb 2B11 did not bind 2B11‐captured C5 as anticipated, but all the other mAbs bound C5 captured on 2B11, demonstrating no competition between 2B11 and any of the other in‐house or commercial mAbs. (B) When mAb 4G2 was used as capture, mAb 4G2 as detect did not bind, but all other mAbs bound 4G2‐captured C5, demonstrating no competition between 4G2 and any of the other in‐house or commercial mAbs. (C) With mAb 7D4 as capture, 7D4 as detect did not bind, but all other mAbs except eculizumab bound captured C5, demonstrating specific competition between mAb 7D4 and eculizumab. Competition was confirmed in the other orientation with eculizumab as capture (F). (D) With mAb 10B6 as capture, 10B6 as detect did not bind but all other mAbs except crovalimab bound captured C5, demonstrating specific competition between mAb 10B6 and crovalimab. Competition was confirmed in the other orientation with crovalimab as capture (E). Results are representative of three independent ELISA experiments, all showing similar results. Error bars are standard error of the mean (SEM) of assay duplicates.

**FIGURE 3:**
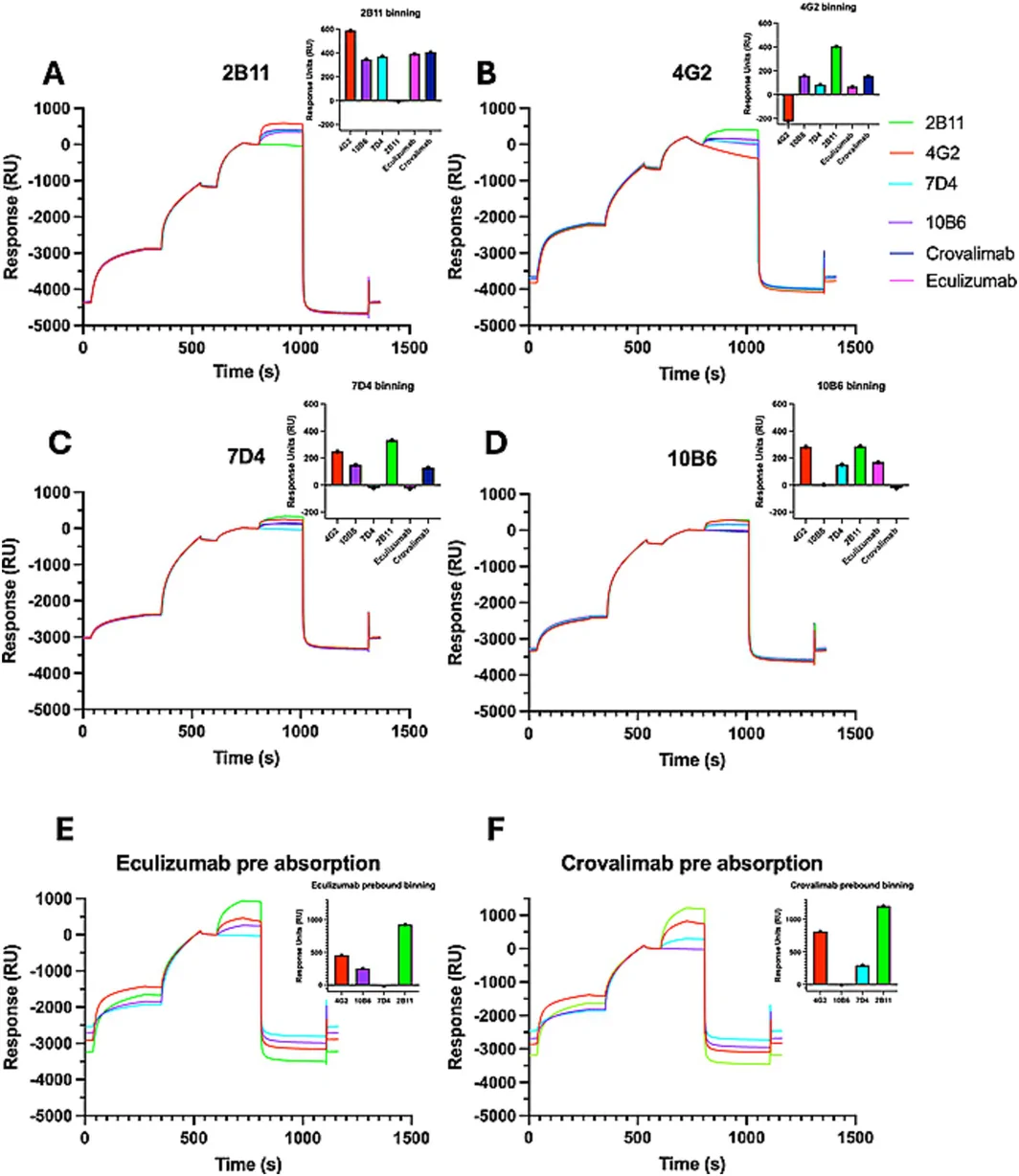
Pairwise epitope binning of anti‐C5 mAbs on human C5 by surface plasmon resonance (SPR). To confirm the competition ELISA results, mAbs 2B11 (A), 4G2 (B), 7D4 (C) and 10B6 (D) were separately captured on an SPR chip using the mouse IgG capture kit (GE Healthcare) at approximately 1400 RU (phase 1 in sensorgram). After capture, the surface was blocked with IgG (phase 2 in sensorgram), and C5 flowed in HEPES buffer at 100 nM to saturate the binding (phase 3 in sensorgram). Individual mAbs were then flowed at 100 nM (10 μL/min) (phase 4 in sensorgram), binding evaluated using Biacore T200 evaluation software, and graphed using Prism10. Between runs, the chip was regenerated using 10 mM glycine, pH 1.7. Binding results replicated those in ELISA with mAb 2B11‐captured and mAb 4G2‐captured C5 binding all other mAbs, while 7D4‐captured C5 did not bind eculizumab and 10B6‐captured C5 failed to bind crovalimab. Inserts in each panel summarise the binding patterns from the SPR analysis. The human mAbs eculizumab and crovalimab were incompatible with the mouse capture kit; to test these, in‐house mAbs were separately captured on SPR chip (phase 1 in sensorgram) and blocked (phase 2 in sensorgram as above). C5 was mixed with an excess of eculizumab (E) or crovalimab (F) prior to flowing over each immobilised mAb for the analysis (phase 3 in sensorgram). Eculizumab saturation prevented binding of C5 to 7D4 but not the other mAbs while crovalimab saturation prevented binding of C5 to 10B6 but not the other mAbs, consistent with the findings from competition ELISA. All sensorgrams are representative of three individual experiments showing similar results.

To confirm the presence of four distinct epitopes for C5‐blocking mAbs on C5, a further SPR experiment was conducted. mAb 2B11 was immobilised and used to capture C5; sequentially flowing each of the in‐house mAbs (7D4 then 2B6 then 4G2) demonstrated binding of each mAb to the 2B11‐captured C5, confirming that all four mAbs could simultaneously bind C5 through separate epitopes on the C5 protein (Figure 4). Of note, 7D4 prebinding prevented eculizumab binding while 10B6 prebinding blocked crovalimab (Figure 4). Flowing the capture mAb 2B11 at the end of the experiment showed no signal, confirming binding specificity (Figure 4).

**FIGURE 4:**
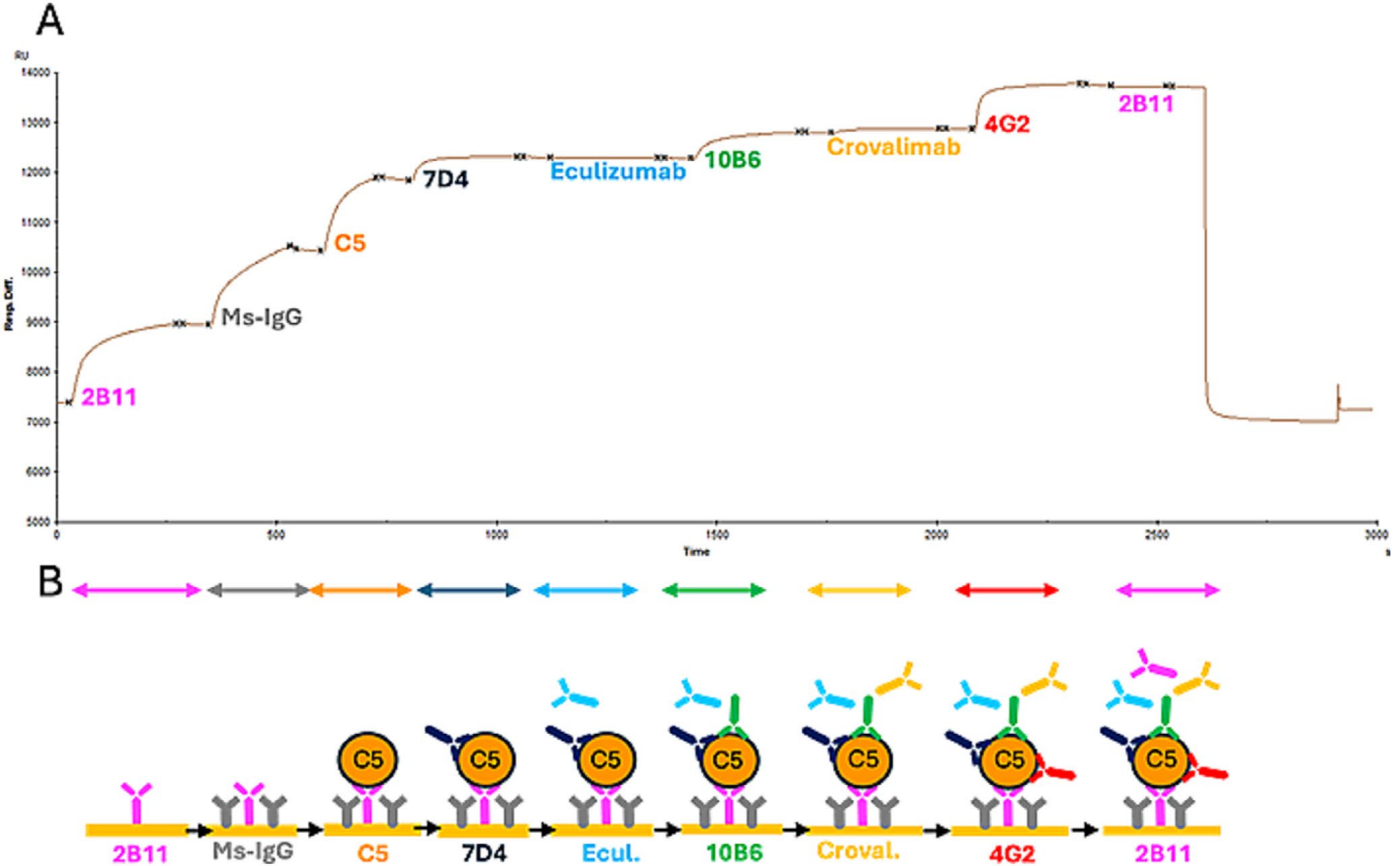
Sequential addition on SPR confirms simultaneous C5 binding of four mAbs. (A) To determine the number of epitopes on C5 that could be simultaneously occupied by the tested anti‐C5 mAbs, 2B11 mAb, not competitive with any other mAb, was immobilised on the chip at ~1800 RU (phase 1 in sensorgram), the chip blocked (phase 2), C5 at 100 nM captured (phase 3) and each of the other mAbs at 100 nM sequentially flowed (10 μL/min) to saturate its binding site on C5. In the example shown, 7D4 bound the immobilised C5 (phase 4), thereby blocking the binding of eculizumab (phase 5); flowed 10B6 then bound the captured C5 (phase 6), blocking the crovalimab epitope (phase 7). Flowed 4G2 bound the captured C5 (phase 8), confirming the simultaneous occupancy of all four unique epitopes. Finally, mAb 2B11 was flowed and failed to bind, as anticipated (phase 9). Sensorgrams are representative of three individual experiments all showing similar results and RU values. (B) A cartoon illustration of the above experiment with colour coding to explain the stepwise process of C5 capture and mAb binding.

### 3.3 mAbs 2B11 and 4G2 Inhibit Haemolysis and Generation of TCC But Not C5a

Both eculizumab and crovalimab are reported to block C5 cleavage, thus preventing both C5a release and TCC/MAC formation; we here tested whether the four novel anti‐C5 mAbs also blocked C5 cleavage. The six mAbs were included in individual CH50 assays at a dose that completely inhibited haemolysis (50 μg/mL; Figure 1B). Supernatants from these assays were then tested in ELISAs to measure C5a and TCC. All six mAbs inhibited generation of TCC, completely except for mAb 4G2, the weakest haemolysis blocker, where residual TCC was detected (Figure 5A). Crovalimab, eculizumab and mAbs 7D4 and 10B6 also inhibited C5a generation (Figure 5B). In contrast, neither 2B11 nor 4G2 inhibited C5a generation at a dose that blocked haemolysis (Figure 5B), demonstrating that they blocked TCC/MAC generation without preventing C5 cleavage.

**FIGURE 5:**
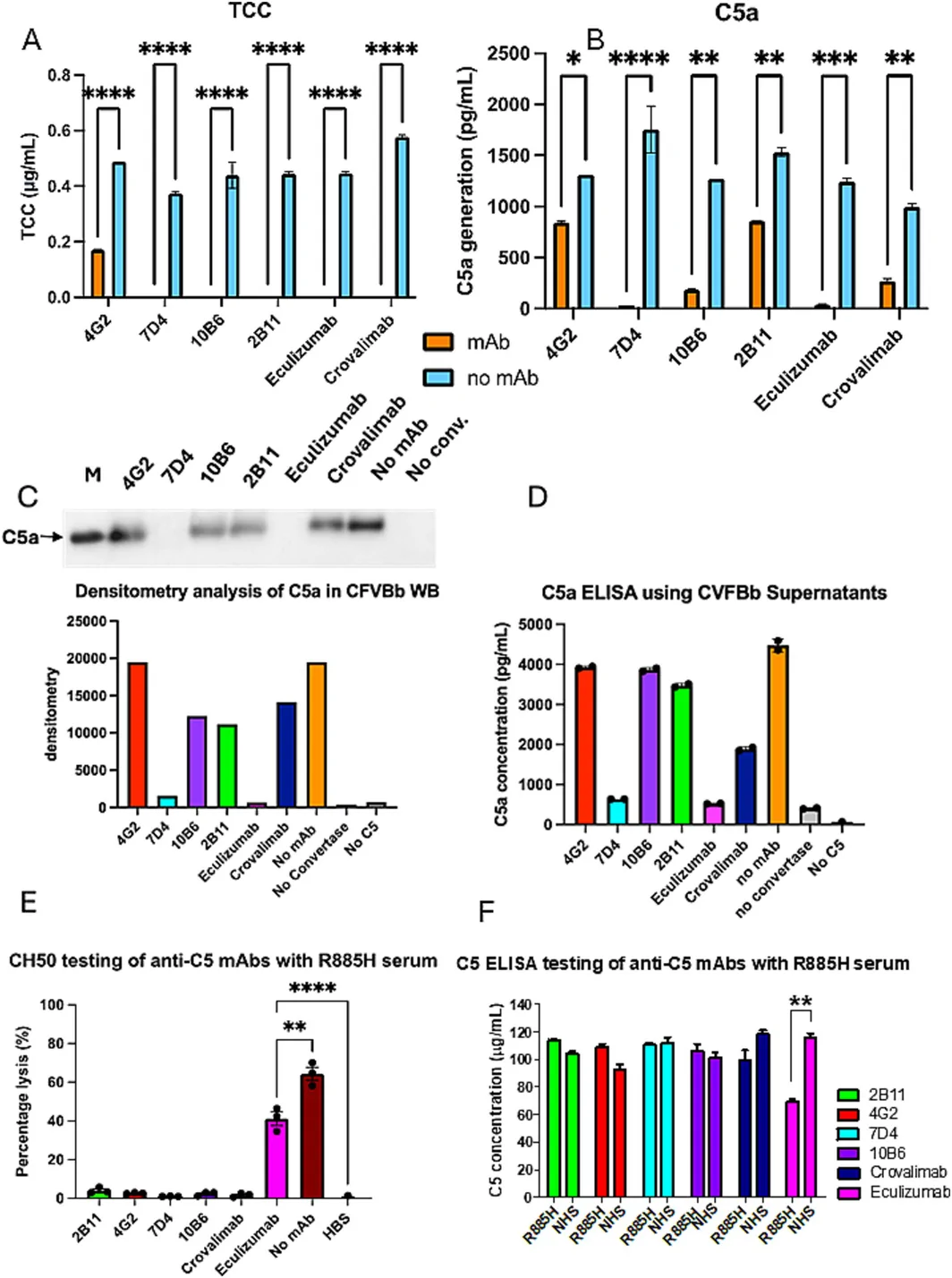
Inhibition of C5 cleavage and binding of R885H C5 by anti‐C5 mAbs. (A and B) Generation of TCC and C5a was measured in supernatants from classical pathway haemolysis (CH50) assays. For each mAb, supernatants were harvested from the CH50 assays (as in Figure 1A) at zero antibody (no inhibition) and 50 μg/mL antibody (maximum inhibition); TCC and C5a in these supernatants were measured by ELISA. All mAbs inhibited TCC generation in the haemolysis assay (A); however, mAbs 2B11 and 4G2 failed to inhibit C5a generation in the assay (B). Two‐way analysis of variance (ANOVA) with Dunnett's multiple comparisons test was used to compare TCC and C5a generation for each mAb with the no mAb control. Data are presented as mean ± SEM. *p < 0.05, **p < 0.01, ***p < 0.001, ****p < 0.0001. (C and D) To test whether the mAbs inhibited C5 cleavage by the CVFBb convertase, C5 was incubated with CVFBb in the presence of the mAbs (2B11, 4G2, 7D4, 10B6, eculizumab, crovalimab; 5:1 M excess). C5a generation in assay supernatants was measured by western blotting with anti‐C5a/C5adesarg mAb, quantified by automated ImageJ densitometric analysis of the C5a band (C) and in the C5a ELISA (D). In both assays, only 7D4 and eculizumab blocked C5a generation. CVFBb convertase efficiently cleaved C5 in the absence of mAb (no mAb) and there was no cleavage in the absence of convertase (no conv). Western blots were representative of three individual experiments yielding similar results; uncropped whole blot and secondary only control are shown in Figure S3E,F. The capacity of each mAb to inhibit complement‐mediated lysis by C5 R885H heterozygous serum was assessed in CH50 assay (E). mAbs were tested at a concentration that completely blocked haemolysis in NHS (100 μg/mL). Eculizumab partially (~50%) reduced haemolysis in R885H serum, while the other mAbs, including the eculizumab‐like antibody 7D4, caused complete inhibition of R885 serum haemolysis. One‐way ANOVA with Dunnett's multiple comparisons test was used to compare each mAb with the no mAb control. Data are presented as mean ± SEM. *p < 0.05, **p < 0.01, ***p < 0.001, ****p < 0.0001. Capacity of the anti‐C5 mAbs to measure C5 in NHS and R885H serum was compared in sandwich ELISA with C5 captured on a polyclonal anti‐C5 and the different mAbs as detect. C5 levels measured using eculizumab as detect were lower by ~46% in R885H serum compared to NHS, while for all the other mAbs, measured levels in R885H serum and NHS were similar. Two‐way ANOVA with Tukey's multiple comparisons test was used to compare haemolysis in R885H serum and NHS for each mAb. Data are presented as mean ± SEM. *p < 0.05, **p < 0.01, ***p < 0.001, ****p < 0.0001 (F).

Impact of the mAbs on C5 cleavage was also tested using the CVFBb C5 convertase. Eculizumab and the eculizumab‐competitive mAb 7D4 completely inhibited cleavage of C5 and release of C5a by the CVFBb convertase, demonstrated by measurement of C5a by densitometry (Figure 5C; uncropped blot in Figure S3) and ELISA (Figure 5D), while all the other mAbs tested, including crovalimab and 10B6, permitted C5a generation by the CVFBb convertase (Figure 5C,D). Crovalimab and 10B6 blocked C5 cleavage and C5a release by native but not CVFBb convertase, likely because of structural and functional differences between the convertases [18].

To further explore C5 epitope specificity and functional differences between the mAbs, capacity to inhibit C5 in serum from a donor heterozygous for the eculizumab‐resistant R885H C5 variant was tested in haemolysis assays at a dose (50 μg/mL for all mAbs) that completely blocked NHS haemolysis. Eculizumab failed to completely block haemolysis in R885H (heterozygous) serum, causing a reduction of ~30% compared to no mAb control; in contrast, all the other mAbs, including the eculizumab‐competitive mAb 7D4, completely blocked haemolysis in R885H serum (Figure 5E). To confirm this finding, the different mAbs were used as detection in a C5 ELISA comparing NHS and R886H serum. While the eculizumab detection assay measured ~46% lower C5 levels in R885H serum compared to NHS, crovalimab and all in‐house anti‐C5 mAbs measured similar levels of C5 in the sera, confirming that they bound C5 R885H (Figure 5F).

### 3.4 mAbs 2B11 and 4G2 Bind C5b6 and Block Recruitment of Downstream Components

To further explore the mechanism by which the novel mAbs 2B11 and 4G2, targeting unique epitopes in C5/C5b6, inhibit reactive lysis and formation of C5b‐9, C5b6 was first captured to the SPR chip via CAP‐immobilised eculizumab. In the absence of added mAbs, sequentially flowing C7, C8 and C9 demonstrated efficient assembly of the C5b‐9 complex (Figure 6A). When C7 was omitted, C8 and C9 did not bind and when C8 was omitted, C9 did not bind (data not shown). In separate experiments, the mAbs 2B11 and 4G2 (100 nM) were flowed over the surface, demonstrating binding to C5b6 and confirming that their binding epitopes are available on eculizumab‐captured C5b6. Sequentially flowing C7, C8 and C9 confirmed that mAbs 2B11 and 4G2 both blocked C7 binding to C5b6 and prevented recruitment of C8 and C9 (Figure 6B,C).

**FIGURE 6:**
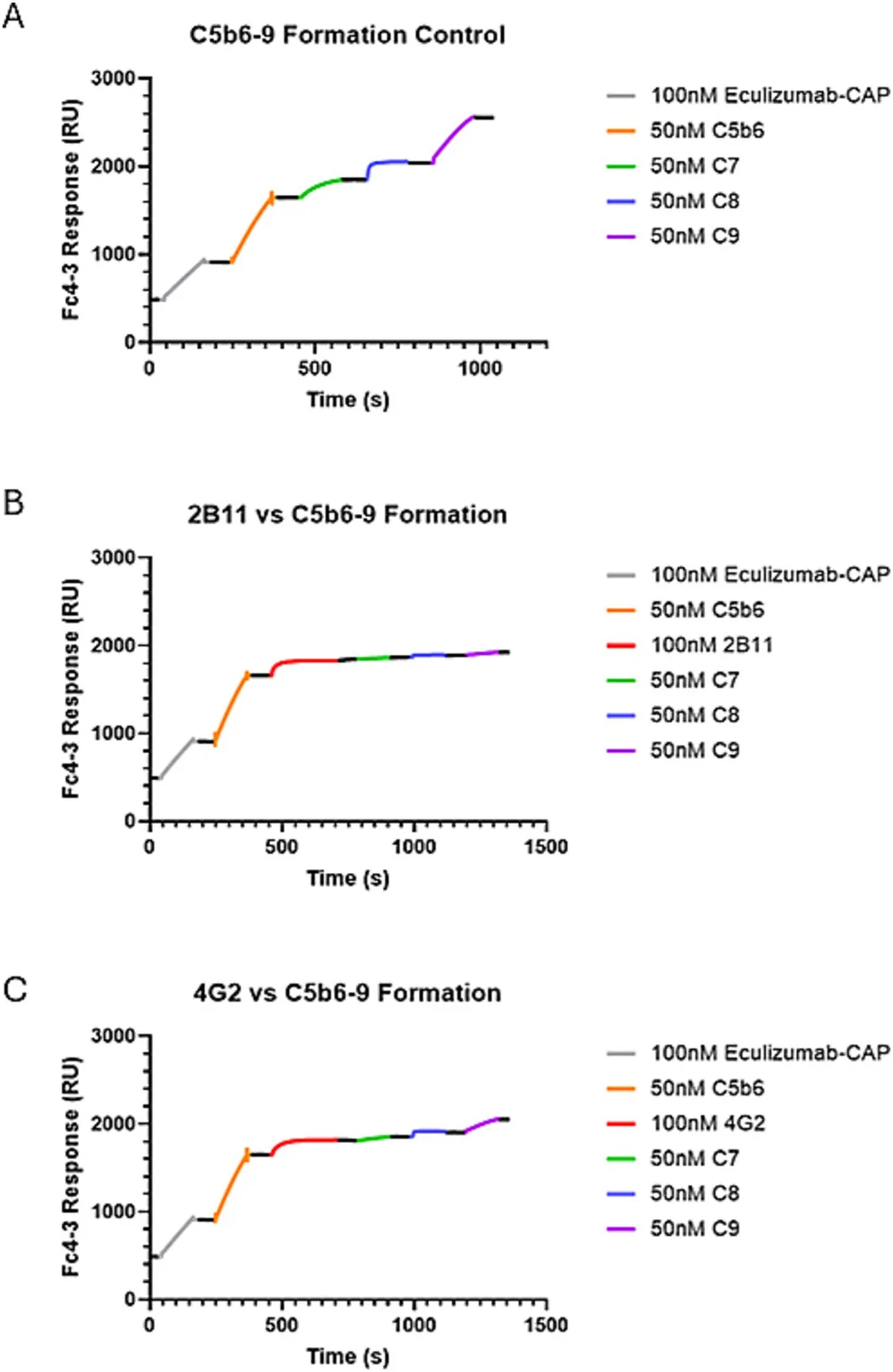
mAbs 2B11 and 4G2 bind C5b6 and block recruitment of downstream components. (A) Baseline formation of C5b‐9 without any blocking mAb intervention. C5b6 was captured on an immobilised eculizumab surface and terminal complement components C7, C8 and C9 sequentially flowed at 50 nM. Clear binding of each component is seen. (B and C) mAbs 2B11 (B) and 4G2 (C) bind to captured C5b6 and block C7 recruitment to C5b6 and subsequent recruitment of C8 and C9.

## 4 Discussion

The C5‐blocking mAb eculizumab received FDA approval for PNH in 2007 [19]. Recently, several other C5‐targeting complement therapeutics have been approved, including mAbs (crovalimab, ravulizumab), peptides (zilucoplan) and RNA aptamers (avacincaptad pegol) [1, 5, 20, 21, 22]. Preclinical C5 cleavage‐blocking nanobodies have also been reported [23, 24]. The newer anti‐C5 mAbs include modifications to improve half‐life; for example, ravulizumab (FDA approved 2018), derived from eculizumab, incorporates antibody recycling to extend the dosing interval [5], while crovalimab (FDA‐approved 2024) includes pH switch recycling technology (SMART) [8]. Eculizumab/ravulizumab bind the MG7 domain in C5α chain [7, 25, 26]; the epitope includes R885 and the variant R885H (~3% frequency in Japan) confers eculizumab/ravulizumab resistance [11, 27]. Crovalimab binds the C5β chain binding and is unaffected by this variant [11, 28, 29]. Both eculizumab and crovalimab prevent C5 convertase cleavage, inhibiting both C5a release and generation of C5b to seed terminal pathway activation [30].

The demonstration of two distinct function‐blocking epitopes on C5 led us to question whether there were other blocking epitopes revealing alternative inhibitory mechanisms. Although previous studies, including those describing the tick‐derived inhibitor OmCI and RaCI, have demonstrated that C5 inhibition can occur through mechanisms other than direct blockade of the convertase cleavage site, [31, 32] whether additional functional blocking epitopes on C5 exist has not been explored. We compared epitopes and mechanisms of four in‐house C5‐blocking mAbs with commercial mAbs; three mAbs (4G2, 7D4, 10B6) were described previously but not epitope‐mapped [10], while one (mAb2B11) was not previously reported. All the mAbs bound C5 and inhibited complement haemolysis with similar efficacy. Competition ELISA and SPR epitope binning showed that mAb7D4 competed with eculizumab for C5 binding (epitope 1) while mAb10B6 competed with crovalimab (epitope 2). The other two in‐house mAbs (2B11, 4G2) bound a unique site on C5, epitopes 3 and 4 respectively. The eculizumab epitope (epitope 1) on C5 resides entirely within the MG7 domain [7]. Although eculizumab and 7D4 compete for the same epitope, the former is human‐specific while the latter potently inhibits rat C5 [10], implying that they bind overlapping/adjacent but distinct epitopes in MG7. Key MG7 binding residues are conserved between human and rat except for a W > S substitution at residue 917, implicated in the species specificity of eculizumab [33]. The second competing pair (epitope 2) comprises crovalimab and 10B6. Crovalimab binds the C5 MG1 domain, encompassing β‐chain residues 20–124 [28]. Our previously published data show that 10B6 is human‐specific while crovalimab shows cross‐species inhibitory activity, including for mouse C5, implying overlapping but nonidentical epitopes for these mAbs [9, 10]. Simultaneous C5 binding of eculizumab and crovalimab was demonstrated in drug switchover studies [34]. We show using ELISA and SPR that all four distinct blocking epitopes on C5 can be occupied simultaneously.

Neither eculizumab nor crovalimab bind C5 at the convertase scissile bond yet both prevent C5 cleavage and C5a release, ascribed to steric hindrance and/or conformational locking of C5 [31, 35]. The mAbs 7D4 (eculizumab‐competitive) and 10B6 (crovalimab‐competitive) also blocked C5 cleavage and C5a release. In contrast, mAbs 2B11 and 4G2, binding distinct C5α chain epitopes, permitted C5 cleavage and C5a release despite efficiently inhibiting haemolysis, demonstrating a distinct inhibitory mechanism. To explore this mechanism, all mAbs were tested in reactive lysis assays, added either before C5b6, after C5b6 but before C7 and after adding C7 to form C5b‐7; eculizumab and mAb 7D4 failed to prevent lysis when added at any stage of the assay, demonstrating that these C5 cleavage blockers were inactive in reactive lysis as previously noted for eculizumab [17]. The C5 cleavage blockers crovalimab and mAb 10B6 shared a second inhibitory mechanism, inhibiting in reactive lysis when added either before or after incubation of C5b6 with GpE, but not after addition of C7, demonstrating that they bind not only C5 but also C5b in the C5b6 complex to prevent C7 binding and MAC assembly; the mAbs 2B11 and 4G2 inhibit solely by this latter mechanism.

To interrogate functional differences, the mAbs were tested for inhibition of CVFBb‐mediated C5 cleavage; only eculizumab and mAb7D4 efficiently inhibited C5 cleavage, indicating that inhibition of C5 cleavage differs between the native C5 convertase and the CVFBb convertase. The mechanistic basis for this difference remains unclear and warrants further investigation. Other mAbs either partially inhibited (crovalimab) or failed to inhibit (10B6, 2B11, 4G2) CVFBb‐mediated C5 cleavage, implying subtle differences in the requirements for C5 cleavage by the native convertase and the CVFBb convertase. Similar findings were reported for the tick‐derived inhibitor OmCI, inhibiting C5 cleavage by the native convertase but not CVFBb [31, 32]; OmCI engages the C5 MG8 domain overlapping the C5 convertase cleavage site [31, 32]. Here we identified four function‐blocking epitopes on C5, including two previously unrecognised epitopes that permit C5 cleavage and C5a generation while selectively preventing MAC assembly.

We next tested the capacity of the different mAbs to bind and inhibit the R885H C5 variant, resistant to eculizumab inhibition [11, 27]. While eculizumab failed to inhibit haemolysis in R885H heterozygous serum at a dose that ablated lysis in normal serum, the eculizumab‐competitive mAb7D4 and all the other mAbs tested efficiently inhibited C5 R885H donor serum. Comparison of measured levels in R885H serum and NHS in sandwich ELISAs confirmed that while eculizumab did not bind C5 R885H (measured levels ~50% of NHS) while crovalimab and all four in‐house mAbs measured similar C5 levels in NHS and R885H serum. These findings confirm our mapping data, mAb7D4 and eculizumab bind similar/overlapping epitopes but with key differences; R885, essential for eculizumab binding [7, 11, 33], is not required for binding of 7D4. This observation has clinical relevance in that 7D4 offers an eculizumab analogue that circumvents eculizumab treatment resistance in R885H carriers.

Of the four C5‐blocking mAbs described here, 7D4 and 10B6 mimic current therapeutic mAbs eculizumab and crovalimab respectively, blocking C5 cleavage, C5a generation and MAC/TCC formation. The mAbs 2B11 and 4G2 are noncompetitive with these and block MAC formation without preventing C5 cleavage and C5a generation, providing new tools to dissect the impacts of inhibiting C5a and MAC in disease contexts and new routes to therapy. The demonstration that the mAbs 2B11 and 4G2 block C5b‐9 complex formation measured in real time using SPR supports our interpretation of the reactive lysis data and highlights a key difference between these mAbs targeting novel epitopes in C5 and the clinical C5‐blocking mAbs eculizumab and crovalimab. In diseases like PNH, where MAC‐mediated haemolysis is the core feature, inhibition of MAC alone will be just as effective as current mAb therapeutics blocking MAC and C5a generation. Given that C5a generation may have protective roles in disease contexts, for example, as a cytoprotective and host defence mechanism [36, 37], drugs that specifically block MAC may have therapeutic advantages and reduce risk of infections. Testing these different C5‐targeting modalities in relevant disease models will help further clarify the relative benefits of blocking C5 cleavage (thereby inhibiting both C5a and MAC) versus selective inhibition of MAC formation, and define the optimal therapeutic approach for different disease settings. Indeed, we have previously demonstrated the efficacy of the MAC‐selective mAb 4G2 in experimental myasthenia gravis, highlighting the therapeutic potential of this approach while emphasising the need for further validation across additional disease models [15].

## Author Contributions

R.S.C., K.Y.L. and W.M.Z. performed all laboratory analyses. W.M.Z. generated the in‐house mAbs. R.S.C. wrote the first draft of the manuscript. M.M. donated the R885H donor serum. B.P.M. and W.M.Z. conceived and designed the study and oversaw data handling and manuscript preparation. All authors contributed to the article and approved the final submitted version.

## Funding

This work was supported by the UK Research and Innovation (526567), the UK Dementia Research Institute (UK DRI‐3002) and the Alzheimers Society (565).

## Conflicts of Interest

The authors declare no conflicts of interest.

## Supporting information


**Figure S1:** Confirmation of C5 subunit binding by the mAbs. Western blot (WB) of human C5 (1 μg/well), nonreduced or reduced, probed with mAbs 2B11 (A), 4G2 (B), 7D4 (C), 10B6 (D), eculizumab (E) and crovalimab (F). All mAbs detected nonreduced C5 at the anticipated MW. On reduction, mAbs 2B11, 4G2, 7D4 and eculizumab bound the C5α chain, while mAb 10B6 and crovalimab bound the C5β chain. Results are representative of at least three analyses.

**Figure S2:** Kinetic analysis of 2B11 using surface plasmon resonance. Binding kinetics of 2B11 to human C5 had not previously been determined. C5 was immobilised on a CM5 chip (RU = 8400) and mAb flowed over (10 μL/min) in a series of concentrations (50–0 nM) as illustrated. Sensorgrams were collected and *K*_D_s calculated using the Langmuir 1: 1 binding model with RI values set to zero. Calculated binding parameters were: ka = 6.08 × 10^−5^ (1/Ms), kd = 3.347 × 10^−4^ (1/s) KD = 5.505 × 10^−10^ (M). A representative sensorgram is shown with raw data in coloured lines and fitted data in black (*n* = 3). Data are representative of three individual experiments all showing similar RU values.

**Figure S3:** Uncropped blots of CVFBb western blot. Whole membranes for the Western blot referred to in Figure 5C. Briefly, preincubated C5‐mAb complexes were incubated with CVFBb for 1 h at 37°C and subject to analysis by WB under reducing conditions. Primary mAb at 2 μg/mL (C5a/C5a des‐Arg clone 2942, Hycult, #H2078; Uden, Netherlands) was added overnight at 4°C, washed in PBS‐T then incubated for 1 h at RT with donkey antimouse IgG‐HRP (Jackson ImmunoResearch, #715‐035‐150) diluted 1:10 000 in 5% BSA‐PBS‐T. As expected cross reactivity is present with the in house mAbs and the secondary antibody in the control blot, noticeably at 55 and 25 Kda indicative of the IgG heavy and light chain respectively. Importantly, secondary only blot shows no signal at 15 Kda (red box) where C5a is detected.

## Data Availability

The data that support the findings of this study are available from the corresponding author upon reasonable request.
